# Supplementary material for: Cell behaviors underlying Myxococcus xanthus aggregate dispersal
Source: mSystems. 2023 Sep 25;8(5):e00425-23. doi: 10.1128/msystems.00425-23 (PMC10654071; doi:10.1128/msystems.00425-23)
Supplement: Figure S1 — Measures of the cell behaviors that drive aggregation. [file msystems.00425-23-s0001.pdf]

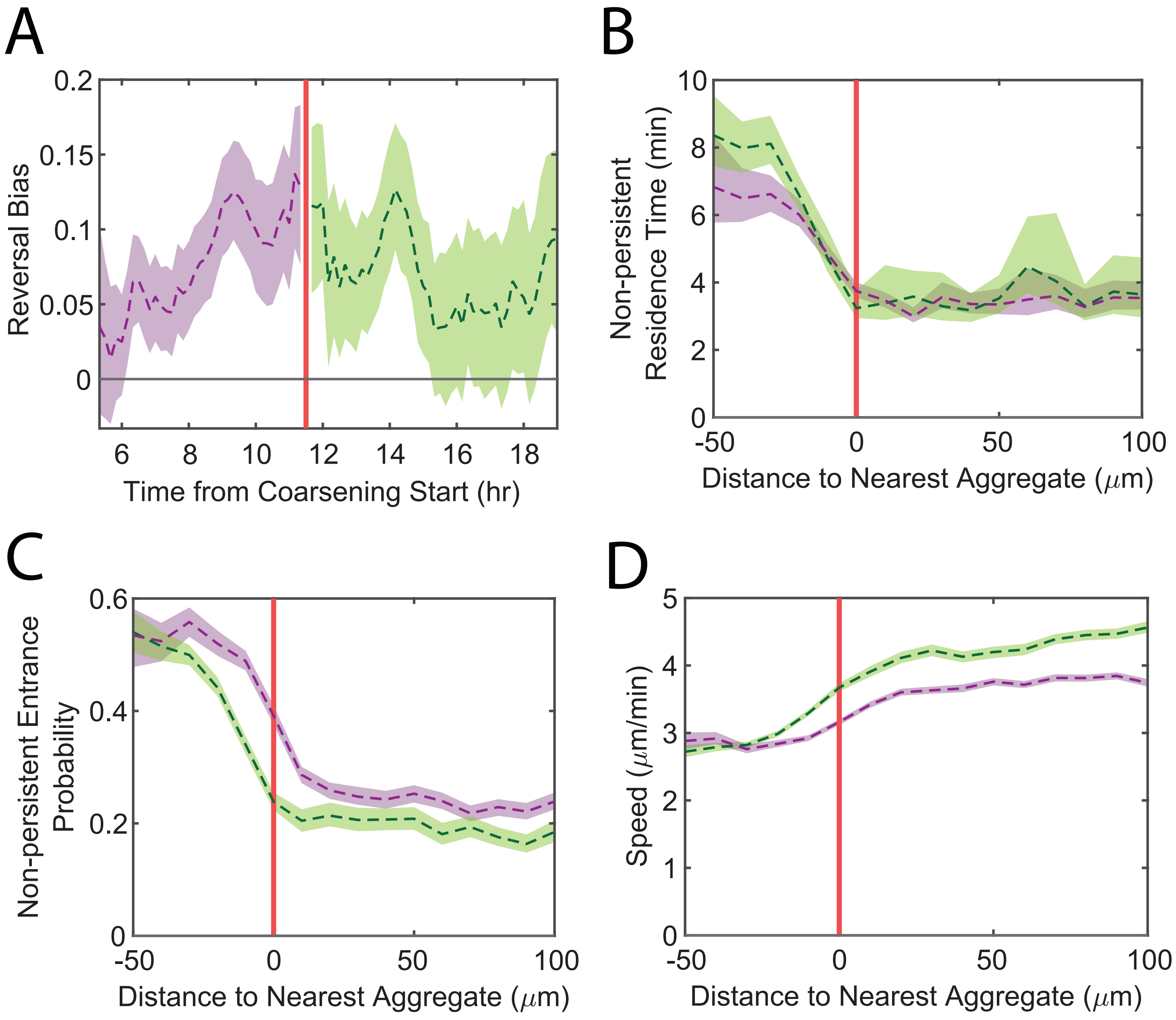

Fig. S1. Measures of the cell behaviors that drive aggregation. A) Mean reversal bias as a function of time, averaged over the field of view. Purple indicates initial aggregation while green indicates the coarsening phase. Note it remains positive throughout. B) Non-persistent state residence time versus distance from the nearest aggregate boundary, averaged over time. C) Non-persistent state entrance probability versus distance from the nearest aggregate, averaged over time. D) Mean speed in the persistent state versus distance from the nearest aggregate boundary, averaged over time. Shaded areas indicate 95% confidence intervals for the mean in all figures. Purple indicates initial aggregation while green indicates the coarsening phase. In B)-D), the red vertical line marks the aggregate boundary.
