# Supplementary material for: Cell behaviors underlying Myxococcus xanthus aggregate dispersal
Source: mSystems. 2023 Sep 25;8(5):e00425-23. doi: 10.1128/msystems.00425-23 (PMC10654071; doi:10.1128/msystems.00425-23)
Supplement: Figure S2 — Reversal bias. [file msystems.00425-23-s0002.pdf]

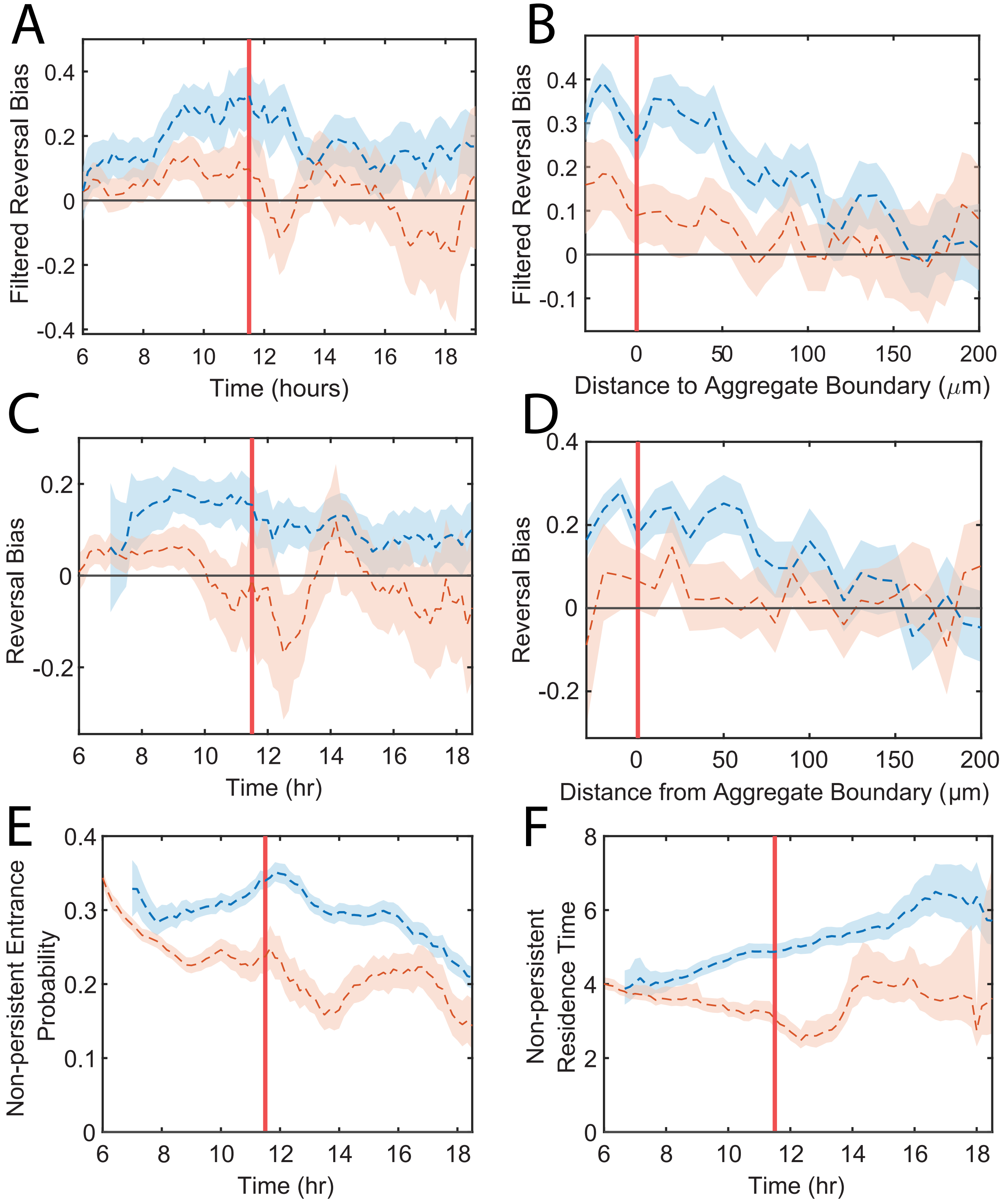

Fig. S2. A) Reversal bias for cells near large (blue) and small (red) aggregates versus time with cells oriented 45-135 degrees in magnitude from the nearest aggregate removed. B) Reversal bias versus the distance from the nearest aggregate's boundary (set to be 0) for cells near stable (blue) and unstable (red) aggregates in the experiment. Like in A), cells oriented 45-135 degrees in magnitude from the nearest aggregate have been removed. C) Plot of the reversal bias for cells near large (blue) and small (red) aggregates versus time. D) Reversal bias for cells near large (blue) and small (red) aggregates versus the distance from the nearest aggregate's boundary (marked by 0). E) The probability of a persistent cell entering a non-persistent state instead of reversing direction of motion for cells near large (blue) and small (red) aggregates versus time. F) Non-persistent state residence time for cells near large (blue) and small (red) aggregates versus time. All plots qualitatively match their equivalents in Figure 3 in the main text, where the same measurements were instead split by nearest aggregate stability rather than size. Large and small aggregates were determined by splitting the aggregates into two populations based on the second quartile of the distribution of aggregate areas.
