# Supplementary material for: Cell behaviors underlying Myxococcus xanthus aggregate dispersal
Source: mSystems. 2023 Sep 25;8(5):e00425-23. doi: 10.1128/msystems.00425-23 (PMC10654071; doi:10.1128/msystems.00425-23)
Supplement: Figure S3 — Reversal bias. [file msystems.00425-23-s0003.pdf]

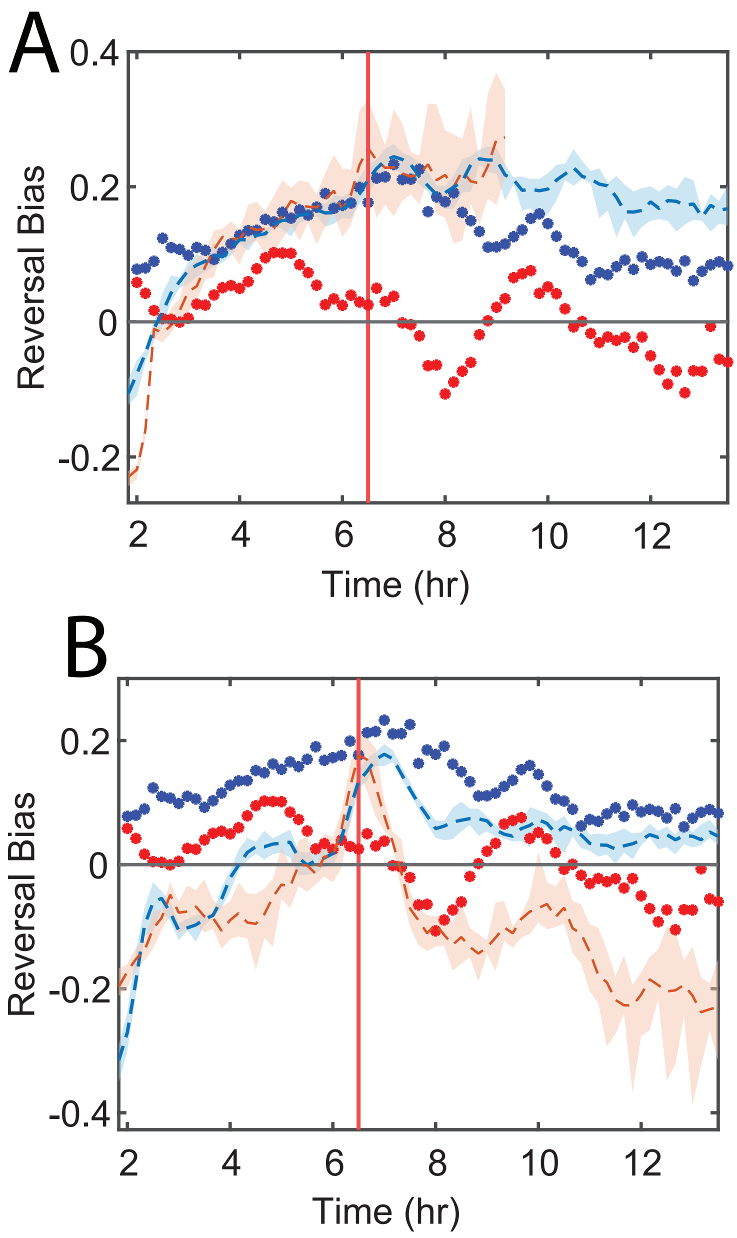

Fig. S3. A) Plot of the reversal bias for stable (dashed blue line) and unstable (dashed red line) aggregates in a simulation using data set 1 with no area-based cue. Blue and red markers indicate the experimental reversal bias for stable and unstable aggregates respectively and the equivalent point in time. B) Same as A) but for a simulation with area-based reversal bias. In both figures, the means were calculated using a 60-minute moving window, vertical red lines mark the start of the coarsening phase, and shaded areas indicate 95% confidence intervals for the mean. In the area-independent simulation, agent cells near both the stable and the few unstable aggregates in this simulation have very similar reversal biases, with both trends matching the reversal bias for stable experimental aggregates. The simulated cells near unstable aggregates never produce a negative mean reversal bias.
