# Supplementary material for: Cell behaviors underlying Myxococcus xanthus aggregate dispersal
Source: mSystems. 2023 Sep 25;8(5):e00425-23. doi: 10.1128/msystems.00425-23 (PMC10654071; doi:10.1128/msystems.00425-23)
Supplement: Figure S4 — Reversal bias. [file msystems.00425-23-s0004.pdf]

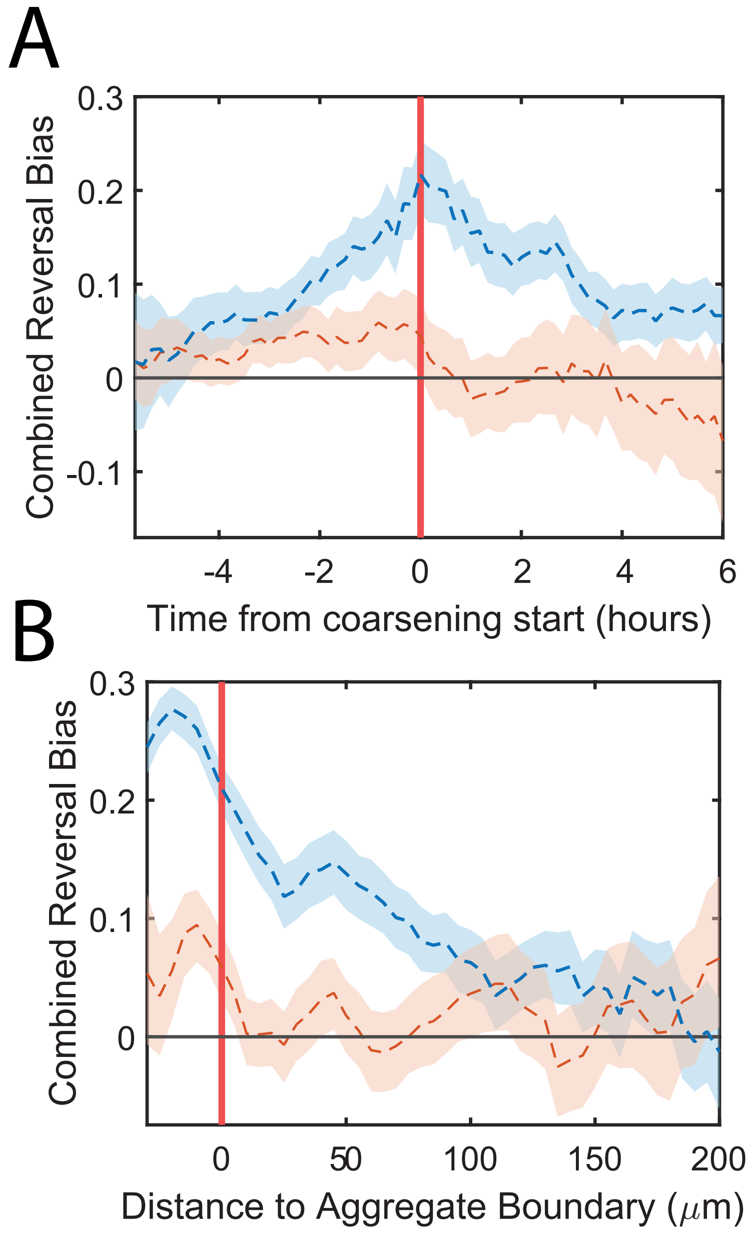

Fig. S4. A) Combined reversal bias from all three HD data sets for cells near large (blue) and small (red) aggregates versus time. Data sets are aligned in time at hour 0 based on the start of the coarsening phase. B) Combined reversal bias from all three HD data sets versus the distance from the nearest aggregate's boundary (set to be 0) for cells near stable (blue) and unstable (red) aggregates in the experiment. Data sets are again aligned in time based on the start of the coarsening phase. The shaded regions in all panels denote 95% confidence intervals for the mean, and the vertical red lines mark the start of the coarsening phase.
