# Supplementary material for: Cell behaviors underlying Myxococcus xanthus aggregate dispersal
Source: mSystems. 2023 Sep 25;8(5):e00425-23. doi: 10.1128/msystems.00425-23 (PMC10654071; doi:10.1128/msystems.00425-23)
Supplement: Figure S5 — Aggregate dispersal. [file msystems.00425-23-s0005.pdf]

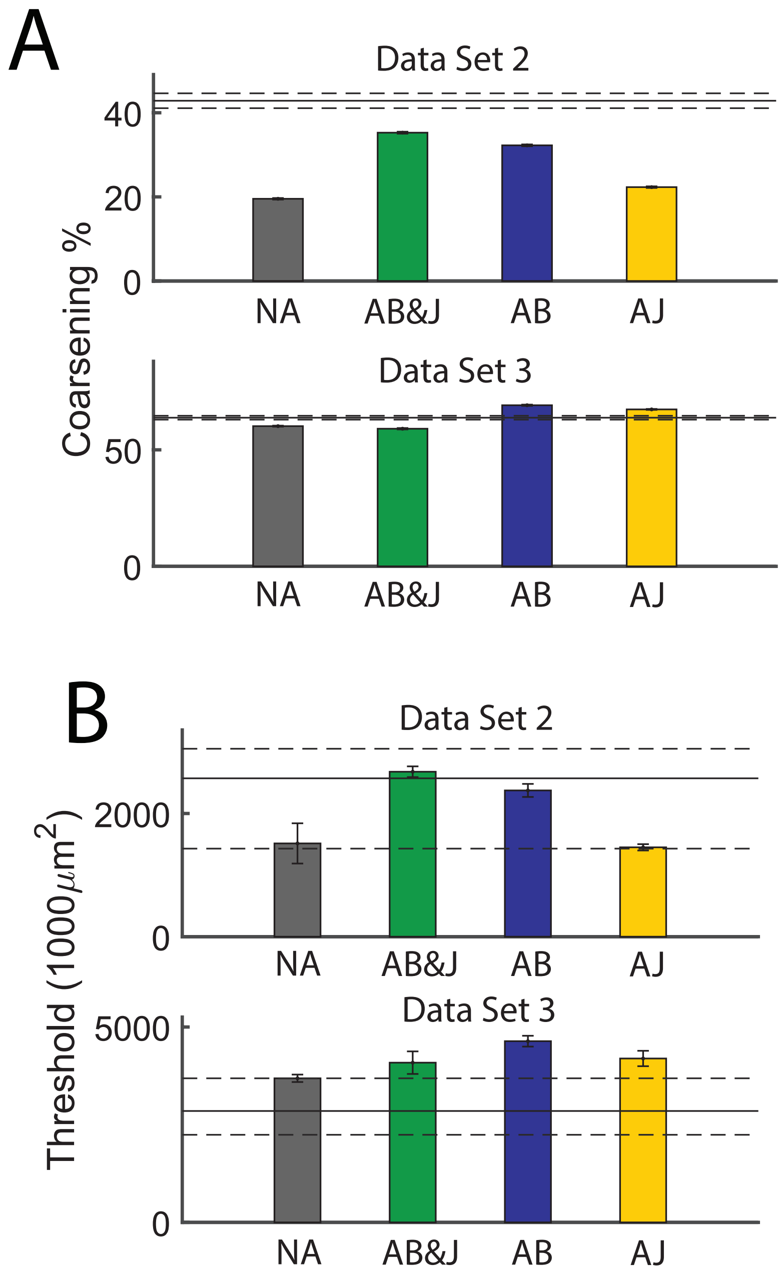

Fig. S5. A) Percentage of aggregates present at the start of coarsening that disperse by the end of the simulation for four different types of simulations: no area cue (NA), both area-based reversal bias and area-based jamming (AB&J), just area-based reversal bias (AB) and just area-based jamming (AJ). Error bars indicated standard error of the mean. The horizonal lines mark the experimental coarsening percentage and its standard error of the mean. For data set 2, the simulations with area-based reversal bias are closest to the experiment, while for data set 3 all simulations produced similar results, with the area-based reversal bias creating the most unstable aggregates. B) Measured size threshold for aggregate dispersal for four different types of simulations: no area cue (NA), both area-based reversal bias and area-based jamming (AB&J), just area-based reversal bias (AB) and just area-based jamming (AJ). Error bars indicated standard error of the mean. The horizonal lines mark the experimental coarsening percentage and its standard error of the mean. For data set 2, the simulations with area-based reversal bias are again closest to the experiment. Likewise, for data set 3 all simulations again produced similar results, with the area-based reversal bias creating having the highest threshold.
