# Supplementary material for: Cell behaviors underlying Myxococcus xanthus aggregate dispersal
Source: mSystems. 2023 Sep 25;8(5):e00425-23. doi: 10.1128/msystems.00425-23 (PMC10654071; doi:10.1128/msystems.00425-23)
Supplement: Figure S6 — Aggregate count. [file msystems.00425-23-s0006.pdf]

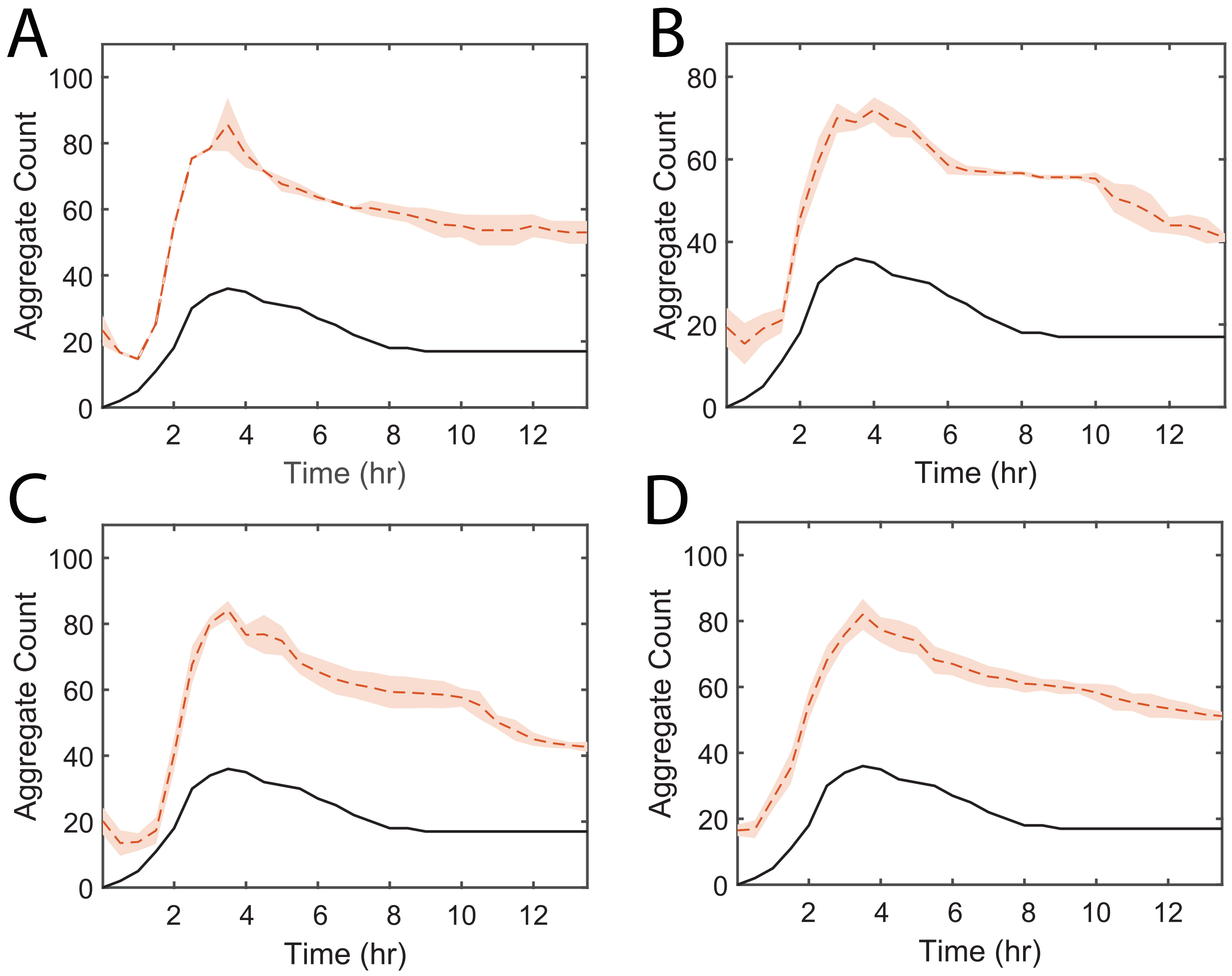

Fig. S6. A)-D) Aggregate count over time for data set 2 experimental results (black) and simulation results (red) when run with no area cue (A), area-based reversal bias and area-based jamming (B), just area-based reversal bias (C) and just area-based jamming (D). Shaded regions mark 95% confidence intervals for the mean. All four types of simulation exhibit increased aggregate formation, with the general trend matching the experiment.
