# Supplementary material for: Cell behaviors underlying Myxococcus xanthus aggregate dispersal
Source: mSystems. 2023 Sep 25;8(5):e00425-23. doi: 10.1128/msystems.00425-23 (PMC10654071; doi:10.1128/msystems.00425-23)
Supplement: Figure S7 — Aggregate count. [file msystems.00425-23-s0007.pdf]

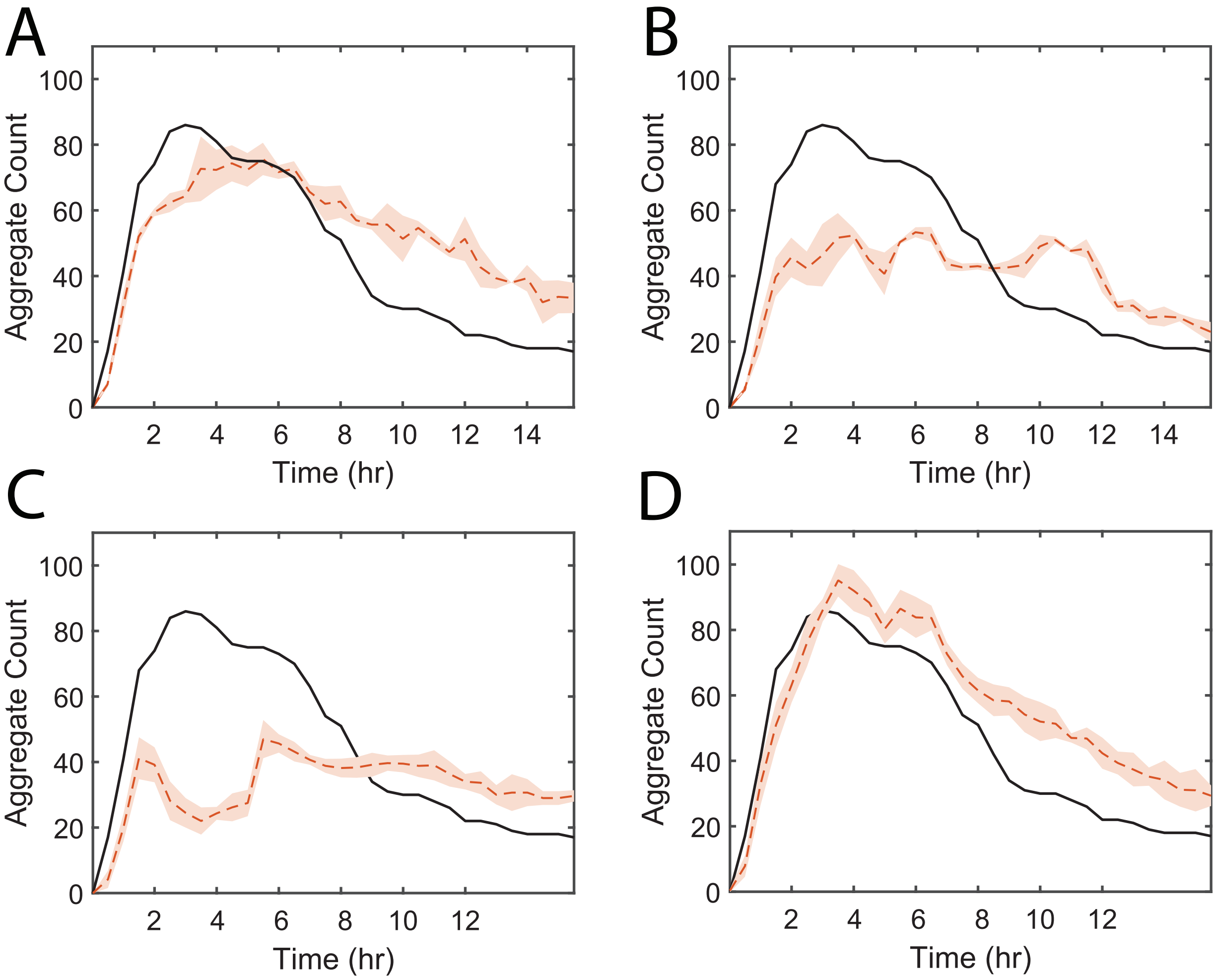

Fig. S7. A)-D) Aggregate count over time for data set 3 experimental results (black) and simulation results (red) when run with no area cue (A), area-based reversal bias and area-based jamming (B), just area-based reversal bias (C) and just area-based jamming (D). Shaded regions mark 95% confidence intervals for the mean. Note all simulation types exhibit aggregate dispersal. The simulations that best matched the experimental trend were those with no area-based reversal bias, with area-based reversal bias alone appearing to trigger an early dispersal phase.
