# Supplementary material for: Cell behaviors underlying Myxococcus xanthus aggregate dispersal
Source: mSystems. 2023 Sep 25;8(5):e00425-23. doi: 10.1128/msystems.00425-23 (PMC10654071; doi:10.1128/msystems.00425-23)
Supplement: Figure S8 — Aggregate count. [file msystems.00425-23-s0008.pdf]

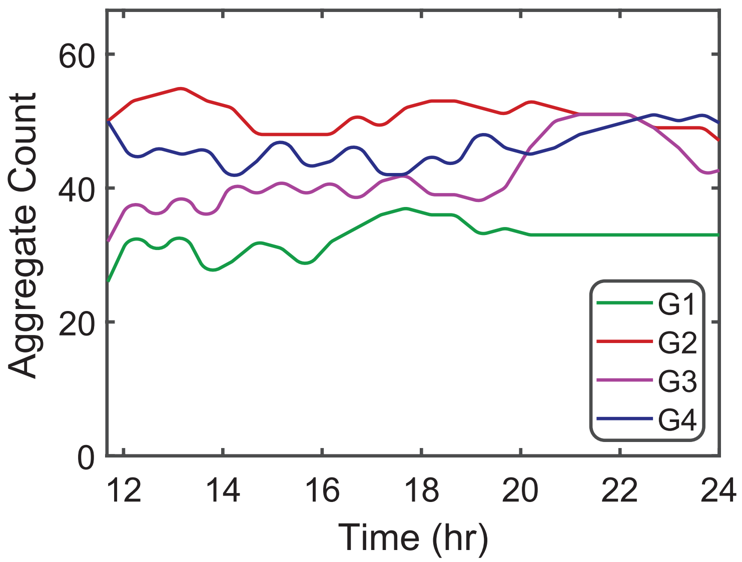

Fig. S8. Aggregate count over time for the four Garza strain replicates G1-G4. Aggregate count is fairly steady over time, with most fluctuations coming from new aggregates forming, merging, or splitting. Dispersal of aggregates is minimal.
